# Use of Chloride of Gold

**Published:** 1869-10

**Authors:** 


Use of Chloride of Gold.—Conheim first demonstrated the termination of the nerves in the cornea, by means of chloride of gold.

